# Diagnosis and Management of Fingolimod-Associated Macular Edema

**DOI:** 10.3389/fneur.2022.918086

**Published:** 2022-07-15

**Authors:** Chunjiang Wang, Zhenzhen Deng, Liying Song, Wei Sun, Shaoli Zhao

**Affiliations:** ^1^Department of Pharmacy, The Third Xiangya Hospital, Central South University, Changsha, Hunan, China; ^2^Department of Endocrinology, The Third Xiangya Hospital, Central South University, Changsha, Hunan, China

**Keywords:** fingolimod, multiple sclerosis, macular edema, visual acuity, vision loss

## Abstract

**Objective:**

To investigate the clinical features, treatment, and prognosis of fingolimod-associated macular edema (FAME) and to provide a reference for its rational management.

**Methods:**

FAME-related case reports were included in a pooled analysis by searching Chinese and English databases from 2010 to November 31, 2021.

**Results:**

The median age of 41 patients was 50 years (range, 21, 67 years), of whom 32 were women. The median time to onset of FAME was 3 m (range.03, 120), and blurred vision (17 cases) and decreased vision (13 cases) were the most common complaints. A total of 55 eyes were involved in FAME, including the left eye (14 cases), right eye (10 cases), and both eyes (15 cases), of which 46 eyes had best-corrected visual acuity close to normal (20/12-20/60) and 8 eyes had moderate to severe visual impairment (20/80-20/500). Fundus examination in 23 patients showed macular edema (11 cases). Optical coherence tomography (OCT) in 39 patients mainly showed perifoveal cysts (24 cases), ME (23 cases), and foveal thickening (19 cases). Fundus fluorescein angiography (FFA) in 18 patients showed vascular leakage (11 cases). Complete resolution of ME occurred in 50 eyes and recovery of visual acuity occurred in 45 eyes at a median time of 2 m (range 0.25, 24) after discontinuation of fingolimod or administration of topical therapy.

**Conclusions:**

Macular edema is a known complication of fingolimod. All patients using fingolimod require regular eye exams, especially those with a history of diabetes and uveitis and those undergoing cataract surgery.

## Introduction

Fingolimod has been approved for the treatment of multiple sclerosis (MS) and relapsing-remitting multiple sclerosis (RRMS) since 2010 (1). Active metabolites of fingolimod bind to the sphingosine-1-phosphate (S1P) receptor on lymphocytes, resulting in internalization and degradation of the receptor. This action prevents the egression of lymphocytes from lymphoid tissue into the circulation, thereby, sparing the central nervous system from attack by myelin-reactive lymphocytes (2).

The most common adverse events reported with fingolimod are nasopharyngitis, bradycardia, influenza, and headache. Macular edema (ME) is a rare complication of fingolimod. Two-phase 3 clinical trial studies have shown that fingolimod is associated with the development of ME (3, 4). Randomized controlled trials have demonstrated that macular edema is related to fingolimod. The current understanding of fingolimod-related macular edema is based primarily on case reports. However, the severity, visual symptoms, visual acuity, treatment, and prognosis of fingolimod-associated macular edema (FAME) remain unclear, especially in patients who wish to continue treatment with fingolimod. The purpose of this study was to explore the clinical features of FAME and provide evidence for its prevention, diagnosis, treatment, and prognosis by pooling and analyzing relevant case reports.

## Methods

### Search Strategy

The related literature on fingolimod-associated macular edema was searched in Chinese and English databases from 2010 to December 31, 2021, including the Wanfang database, China Knowledge Network, VIP database, Web of Science, SpringerLink, PubMed, EMBASE, Cochrane Library, and OVID. Retrieval was performed using a combination of subject words and free words, such as fingolimod, macular edema, vision loss, blurred vision, and retina.

### Inclusion and Exclusion Criteria

Inclusion criteria: case reports or case series of FAME were included. Exclusion criteria: reviews, mechanistic studies, randomized controlled trials, animal studies, and duplicate literature were excluded.

### Data Extraction

Two authors independently screened the literature according to the inclusion and exclusion criteria, and then, the group selected the literature to be included in the analysis. A self-designed data extraction table was used to extract the following information about the patients: nationality, sex, age, history of ophthalmic disease, the purpose of the medication, application of fingolimod, clinical manifestations, ophthalmic-related examinations, treatment, and prognosis.

The Snellen visual acuity classification was used: near normal vision (20/12–20/60), low vision (20/80–20/1,000), and near blindness (≤ 20/1,000). The low vision category was further divided into moderate visual impairment (20/80–20/160), severe visual impairment (20/200–20/400), and profound visual impairment (20/500–20/1,000) (5).

Descriptive statistical analysis was performed on the extracted data.

## Results

### Basic Information

We included 31 studies according to the inclusion and exclusion criteria (6–36). The median age of the 41 patients was 51 years (range 21, 67), including 32 women and 9 men (Table 1). Fingolimod was used to treat MS in 19 patients, RRMS in 21 patients, and renal allograft in 1 patient. Fingolimod was administered at 0.5 mg (30 cases), 5 mg (1 case), 1.25 mg (3 cases,) and not described (7 patients). The median time to the occurrence of MS and RRMS was 78 m (range 0.75, 408). Of these patients, 2 had a history of uveitis, 10 had optic neuritis, 6 had cataracts, and 4 had diabetes. Twenty-one MS or RRMS patients were previously treated, including interferon (12 cases), glatiramer acetate (4 cases), natalizumab (4 cases), steroids (5 cases), azathioprine (2 cases), immunoglobulin (1 case), mitoxantrone (1 case), mycophenolate mofetil (1 case), dimethyl fumarate (1 case), bevacizumab (1 case), and plasma exchanges (1 case). The median time to onset of FAME was 3 m (range 0.03, 120). The median time to onset of ME in patients with diabetes or prior uveitis was 1 m (0.3, 42). Three patients restarted fingolimod and had a re-occurrence of macular edema at 10 d, 2, m and 5 m, respectively. Four patients developed FAME after cataract surgery.

**Table 1:** Basic information of the included 41 patients.

**Reference**	**Age/sex**	**Diabetes, ophthalmic history**	**Indication**	**MS prior treatment**	**MS onset time (Months)**	**Dose** **(mg)**	**ME onset time (Months)**	**BCVA** **(OD)**	**BCVA** **(OS)**	**Uni- or bilateral**
Karakosta and Kourentis (6)	58/m	Cataract	MS	Bevacizumab, steroids	168	0.5	120	20/20	20/30	U (OS)
Gallego-Pinazo et al. (7)	47/f	No	MS	No	108	nr	72	20/20	20/40	U (OS)
Turaka and Bryan (8)	52/m	Optic neuritis	RRMS	No	96	0.5	3	20/20	20/20	U (OS)
Bhatti et al. (9)	54/f	No	RRMS	IFN β-1b	168	0.5	11	20/20	20/80	U (OS)
Thoo et al. (10)	59/f	Optic neuritis	MS	No	0.75	0.5	0.75	6/15	6/12	B
Thoo et al. (10)	66/f	Cataract	RRMS	IFN beta-1b	144	0.5	12	6/12	nr	U (OD)
Li et al. (11)	37/m	Optic neuritis	RRMS	IFN β-1a, mitoxantrone	216	nr	4	6/18	6/18	B
Coppes et al. (12)	60/f	Diabetes	MS	IFNβ, natalizumab, azathioprine	408	0.5	0.3	20/30	20/30	B
Schröder et al. (13)	24/f	Optic neuritis	RRMS	IFN β-1a	24	0.5	1	20/50	20/60	B
Pul et al. (14)	28/f	Diabetes	RRMS	Methylprednisolone	4	nr	1	20/20	20/25	B
Gillam et al. (15)	50/f	Cataract	MS	No	168	0.5	48	6/9	6/12	B
Saab et al. (16)	58/f	No	renal allograft	No	3 m	5	3	20/30	nr	U (OD)
Liu et al. (17)	34/f	Optic neuritis	RRMS	IFNβ-1b	48	0.5	0.17	6/24	6/12	B
Afshar et al. (18)	52/m	No	MS	Mycophenolate mofetil, steroids	nr	0.5	0.23	20/20	20/40	U (OS)
Afshar et al. (18)	60/f	Diabetes, optic neuritis	RRMS	Azathioprine, IFNβ-1b, natalizumab	nr	0.5	0.3	20/60	20/40	B
Afshar et al. (18)	57/m	No	RRMS	IFNβ-1b, IFNβ-1a, glatiramer acetate	nr	0.5	1	20/20	20/30	U (OS)
Chui et al. (19)	67/f	No	RRMS	No	nr	0.5	6	6/7.5	6/6	U (OD)
Minuk et al. (20)	58/f	Uveitis	RRMS	Natalizumab	nr	nr	2	6/15	6/18	B
Asensio-Sánchez et al. (21)	31/f	nr	MS	No	24	0.5	4	20/100	20/50	B
Gaskin and Coote (22)	57/f	Cataract	RRMS	No	24	0.5	24	20/30	20/30	B
Ueda and Saida (23)	31/m	No	RRMS	Steroid, plasma exchanges, LG, IFN β	156	nr	1	20/600	20/500	U (OS)
Akiyama et al. (24)	66/f	Diabetes	RRMS	Prednisolone, IFN β-1b	240	0.5	0.5	nr	nr	U (OS)
Cifuentes-Canorea et al. (25)	56/f	No	MS	no	240	0.5	0.25	20/33	20/29	B
Jasani et al. (26)	54/f	No	RRMS	IFN β-1a	24	nr	3/0.3*	6/12	nr	U (OD)
Schelenz et al. (27)	45/f	No	RRMS	Natalizumab	24	0.5	1	20/20	20/100	B
Khimani et al. (28)	34/m	No	MS	no	18	0.5	18/2*	20/200	20/25	U (OD)
Soliman et al. (29)	42/m	No	RRMS	IFN β-1a	1132	0.5	0.03	20/20	20/30	U (OS)
Sia et al. (30)	45/f	No	MS	no	60	0.5	60	20/20	20/60	U (OS)
Husmann et al. (31)	38/f	No	RRMS	Glatiramer acetate	nr	0.5	6	20/25	20/20	U (OD)
Camós-Carreras et al. (32)	49/f	No	RRMS	Glatiramer acetate	nr	0.5	84	20/32	20/25	B
Kim et al. (33)	62/f	No	MS	Dimethyl fumarate	nr	nr	2.5	20/40	20/80	B
Cugati et al. (34)	41/f	Uveitis	RRMS	Glatiramer acetate	nr	0.5	3	nr	nr	nr
Cugati et al. (34)	66/f	Cataract	RRMS	No	nr	0.5	9	6/12	nr	U (OD)
Zarbin et al. (35)	50/f	Optic neuritis	MS	No	nr	1.25	0.6	20/20	20/40	U (OS)
Zarbin et al. (35)	28/f	Optic neuritis, hyperglycemia	MS	No	nr	0.5	27/5*	20/20	20/40	U (OS)
Zarbin et al. (35)	48/f	Optic neuritis, uveitis	MS	No	nr	1.25	42	20/20	20/28	U (OS)
Zarbin et al. (35)	38/m	Optic neuritis	MS	No	nr	1.25	12	20/20	20/20	B
Zarbin et al. (35)	53/f	No	MS	No	nr	0.5	12	20/40	20/20	U (OD)
Sonne et al. (36)	27/f	No	MS	nr	nr	0.5	5	20/20	20/20	U (OD)
Sonne et al. (36)	65/f	Cataract	MS	nr	nr	0.5	57	nr	nr	nr
Sonne et al. (36)	58/f	No	MS	nr	nr	0.5	9	nr	nr	U (OS)

F, female; M, male; MS, multiple sclerosis; RRMS, relapsing-remitting MS; BCVA, best-corrected visual acuity; OD, right eye; OS, left eye; IFN, interferon; LG, immunoglobulin; nr, not reported. ^*^ Represents the time of ME after taking fingolimod again.

### Clinical Manifestations

A total of 55 eyes were involved in 41 FAME patients, including the left eye (OS) in 14 patients, right eye (OD) in 10 patients, and both eyes (OU) in 15 patients. FAME patients mainly showed blurred vision (17 cases), decreased visual acuity (VA) (13 cases), eye pain (1 case), and asymptomatic vision (3 cases) (Table 2).

**Table 2:** Clinical manifestations, ophthalmic examination, treatment, and prognosis of 41 included patients.

**Reference**	**Symptoms**	**Fundoscopy**	**OCT**	**CFT:OD/OS (μm)**	**FFA**	**Fingolimod**	**Therapy**	**Action outcome**
Karakosta and Kourentis (6)	Blurred vision	Dull foveal reflex with retinal thickening	Foveal cysts and subretinal fluid	-/459	Dot hemorrhages in OU and dye leakage in OS	D	Glatiramer	3 m
Gallego-Pinazo et al. (7)	Visual loss	Branch retinal vein occlusion	Macular thickening with intraretinal cystic spaces	-/396	Intraretinal hemorrhages, delayed choroidal filling	D	Ranibizumab	3 w
Turaka and Bryan (8)	Blurred vision	Clinical ME	CME	280/502	Leakage in the macula of OS	D	Prednisolone	3 m
Bhatti et al. (9)	Visual loss	Retinal hemorrhage involving the fovea with adjacent hard exudate	Hyperdense opacity	nr	Blocked fluorescence by the macular hemorrhage	D	no	3 m
Thoo et al. (10)	Blurred vision	nr	ME	nr	nr	C	Prednisolone, triamcinolone	1 m
Thoo et al. (10)	Visual loss	nr	ME	nr	nr	C	Ketorolac, prednisolone, triamcinolone	2 m
Li et al. (11)	No	Altered foveal light reflexes	Left foveal cyst	262/308	nr	C	Not resolved after 25 m	nr
Coppes et al. (12)	Blurred vision	nr	Bilateral ME	449/586	nr	D	no	1.2 m
Schröder et al. (13)	Blurred vision	Bilateral CME	Foveal cystoid edema	465/452	Leakage of telangiectatic	D	Acetazolamide	nr
Pul et al. (14)	Decreased VA	NPDR	CME	264/463	CME in OU	D	Ranibizumab	2y
Gillam et al. (15)	Blurred vision	nr	CME	nr	nr	D	Ketorolac, methylprednisolone	5 m
Saab et al. (16)	Decreased VA	Retinal pigment epithelium changes with thickening centrally	Macular thickening in CME	nr	CME	D	no	2 m
Liu et al. (17)	Blurry vision	Bilateral CMO with subretinal fluid	CME with subretinal fluid	nr	nr	D	Ketorolac, prednisolone, minimal CME after 5 m	nr
Afshar et al. (18)	Blurred vision	nr	CME	-/573	nr	C	Nepafenac, prednisolone	1 m
Afshar et al. (18)	Blurred vision	CME	CME	477/389	CME in OU	D	no	1 m
Afshar et al. (18)	Blurred vision	CME	CME and an incidental new epiretinal membrane	-/452	nr	D	Bromfenac, blurred vision resolved after 3 months	nr
Chui et al. (19)	Decreased VA	Macular cystic changes	Macular cystic changes	nr	Leakage of dye	D	ketorolac, dexamethasone	5w
Minuk et al. (20)	Decreased VA	nr	CME	506/282	CME	C	Ketorolac, prednisolone, triamcinolone	1 m
Asensio-Sánchez et al. (21)	Decreased VA	CME	CME	nr	nr	D	no	nr
Gaskin and Coote (22)	Blurred vision	CME	CME	573/478	nr	D	DMF, voltaren, prednisolone	1 m
Ueda and Saida (23)	Decreased VA	nr	Retinal hemorrhages and CME	nr	no	D	Betamethasone	24w
Akiyama et al. (24)	No	nr	ME and cystoid appearance	nr	nr	C	no	1y
Cifuentes-Canorea et al. (25)	Blurred vision	nr	CME	480/490	nr	D	no	20d/1 m
Jasani et al. (26)	Blurred vision	nr	Intraretinal cystic fluid spaces	nr	nr	D	DMF, prednisolone, NSAIDS	2 m
Schelenz et al. (27)	Decreased VA	ME	Intraretinal cyst in OD, ME in OS	nr	Leaky and ME in OD, ME in OS	D	DMF, nepafenac, acetazolamide	6 m
Khimani et al. (28)	Blurred vision	nr	Subretinal fluid below right macula	nr	nr	D	DMF	3 m/5 m
Soliman et al. (29)	Blurred vision	Dull foveal reflex with retinal thickening	ME with minimal subretinal fluid	-/613	CME, perivascular leakage of retinal vessels	D	NSAIDS, acetazolamide, dorzolamide, prednisone	nr
Sia et al. (30)	Decreased VA	Retinal hemorrhages and roth spots and macular hemorrhage	Intraretinal hyperreflectivity	nr	Blockage of choroidal fluorescence	C	no	3 m
Husmann et al. (31)	Decreased VA	ME and exudates in right retina	ME in right macula	nr	Leakage of dye	C	Nepafenac	nr
Camós-Carreras et al. (32)	Decreased VA	ME	ME and cystic changes	nr	nr	C	no	4 w
Kim et al. (33)	Blurred vision	nr	ME in OD, CME in OS	325/567	nr	D	Natalizumab, ketorolac, prednisolone	2 m
Cugati et al. (34)	No	nr	nr	nr	nr	D	no	1 m
Cugati et al. (34)	Decreased VA	nr	ME	nr	nr	C	Triamcinolone, NSAIDS, steroid	1w
Zarbin et al. (35)	Eye pain, blurred vision	ME	Increase in CFT	189/416	Petaloid pattern of dye leakage	D	no	65 d
Zarbin et al. (35)	No	nr	Increase in CFT	180/212	Perifoveal dye leakage	D	NSAIDS, steroids.	6w
Zarbin et al. (35)	No	ME	nr	185/276	Perifoveal dye leakage	D	Acetazolamide, steroids	35d
Zarbin et al. (35)	Blurred vision	nr	Increase in CFT	356/341	Perifoveal dye leakage	D	no	2 m
Zarbin et al. (35)	Symptomatic	Intraretinal hemorrhage, branch retinal vein occlusion	Increase in CFT	217/149	ME	D	no	nr
Sonne et al. (36)	nr	Subtle reddish lesion just superior to the fovea	Hyperreflectivity of the outer plexiform layer, filling defect	normal	nr	C	Topical steroids, NSAIDs	5 m
Sonne et al. (36)	nr	nr	nr	nr	nr	C	Topical and sub tenons steroids	nr
Sonne et al. (36)	nr	nr	Normal	nr	nr	C	Bromfenac topical drops	6w

*VA, visual acuity; OCT, optical coherence tomography; OD, right eye; OS, left eye; OU, both eyes; ME, macular edema; CME, cystoid macular edema; CSCR, central serous chorioretinopathy; FFA, fundus fluorescein angiography; CFT, central foveal thickness; NPD, non-proliferative diabetic retinopathy; DMF, dimethyl fumarate; NSAIDS, non-steroidal anti-inflammatory drugs; nr, not reported*.

### Eye Examinations

Among the 55 eyes, 46 eyes had near-normal visual acuity (20/12-20/60), 5 eyes had moderate visual impairment (20/80-20/160), 2 eyes had severe visual impairment (20/200-20/400), and 1 eye had profound visual impairment (20/500-20/1000). The worst visual acuity reported for FAME patients was 20/500 (Table 1). Fundus examination in 23 patients mainly showed macular edema (11 cases), a dull foveal reflex with retinal thickening (4 cases), retinal hemorrhage (3 cases), and exudates (3 cases). Optical coherence tomography (OCT) in 39 patients mainly showed perifoveal cysts (24 cases), ME (23 cases), central foveal thickness (19 cases), subretinal fluid (4 cases), and hyperdense opacity (2 cases). Fundus fluorescein angiography (FFA) in 18 patients showed vascular leakage (11 cases), cystoid macular edema (6 cases), and choroidal filling (3 cases).

### Treatment

Fingolimod was discontinued in 28 patients and continued in 13 patients (Table 2). Seven of 24 patients, who discontinued fingolimod, were switched to glatiramer (1 case), natalizumab (2 cases), and dimethyl fumarate (4 cases). For ME, 17 patients received no treatment, and 25 patients received therapy, including steroids (18 cases), non-steroidal anti-inflammatory drugs (NSAIDs) (11 cases), ketorolac (7 cases), acetazolamide (4 cases), and ranibizumab (2 cases). ME completely resolved in 52 eyes at a median time of 2 m (range 0.25, 24). However, ME did not fully resolve in 3 eyes, and visual recovery was incomplete in 8 of 53 eyes.

## Discussion

The incidence of FAME appeared to be dose-dependent, occurring in 0.4% of patients receiving 0.5 mg and 1% of patients receiving 1.25 mg (37). Our study found that FAME occurred mainly 3 m after the initiation of fingolimod, consistent with the phase III MS study and their extensions (14). However, a small proportion of patients develop late-onset ME (more than 12 months) (6, 15, 24, 30, 35), and blurred vision has also been reported as early as 24 h after initiation of the 0.5 mg dose (29). Patients with ME with diabetes or previous uveitis appear to develop it earlier. FAME cases can be unilateral or bilateral and can present with blurred vision, decreased vision, or no symptoms.

The diagnosis of FAME is based on OCT scanning and FFA to rule out any possible cause of ME. FAME is more likely to occur in patients with a history of diabetes, uveitis, or undergoing cataract surgery (38). Foveal thickening, foveal cysts, subretinal fluid, or dye leakage were seen on OCT scan and FFA in patients with FAME. In addition, ME associated with uveitis due to MS should also be excluded (39).

In addition to having a baseline eye exam before starting fingolimod, the FDA also recommends that patients have an eye exam 3 to 4 months after initiating the drug (35). However, the optimal frequency of follow-up periodic re-evaluation has not been determined, but some researchers recommend regular eye exams after 6 months and then annually (35). Patients who had fingolimod therapy, and who are also undergoing ophthalmic surgery, such as cataract surgery, may be at increased risk of developing ME and may benefit from increased monitoring or preventive treatment for ME (10, 15, 22, 34). Patients who have started fingolimod with a history of diabetes mellitus, uveitis, or concomitant medication associated with ME require closer monitoring.

Although the exact pathogenesis of FAME has not been determined, the likely mechanism is the disruption of the inner blood-retinal barrier. Sphingosine 1-phosphate (SIP) is a biologically active sphingolipid metabolite with five G protein-coupled receptors (S1PR 1-S1PR 5) (40). These receptors are found in nearly every cell type in the body, including lymphocytes, endothelial cells, neurons, and astrocytes. S1PR1 signaling is responsible for maintaining cell-to-cell and cell-to-matrix adhesion. Fingolimod downregulates S1P1R and antagonizes S1P2 R/S1P3R, thus, leading to downregulation of adhesion complexes and increased retinal vascular permeability, resulting in ME (41).

No consensus on best management has yet been established for FAME. Most patients with FAME resolve spontaneously after discontinuation of fingolimod. It should be noted that attempts to restart fingolimod resulted in recurrence of ME, which resolved after discontinuation. For patients requiring continued treatment, topical NSAIDs (e.g., nepafenac, bromfenac, and ketorelac), topical corticosteroids (e.g., prednisolone, and dexamethasone), and carbonic anhydrase inhibitors (e.g., acetazolamide) have been used to accelerate the resolution of FAME. More invasive interventions, such as subconjunctival or intravitreal administration of steroids (triamcinolone) or anti-vascular endothelial growth factor (e.g., ranibizumab and bevacizumab), may be required for refractory FAME (42). Some patients may not respond to topical anti-inflammatory therapy. Intravitreal corticosteroids may be an appropriate treatment option for patients with FAME who are refractory to topical anti-inflammatory therapy (34). However, caution must be exercised whenever steroids are used due to steroid-induced effects, such as ocular hypertension, glaucoma, and cataract formation (43). Patients should be informed of the risks and that they require ongoing monitoring. After the resolution of ME, a small proportion of patients had residual vision loss, which may be related to concurrent cataracts, uveitis, or optic neuritis. In addition, retinal damage is also a possible explanation (42). Determination of whether to discontinue fingolimod therapy should include an assessment of the individual patient's potential benefits and risks and their response to topical anti-inflammatory drugs.

In conclusion, ME is a known complication of fingolimod. FAME usually has a better prognosis with discontinuation or topical therapy. Eye exams are required before and after using fingolimod. Patients undergoing cataract surgery and with a history of diabetes and uveitis require closer monitoring.

## Data Availability Statement

The original contributions presented in the study are included in the article/supplementary material, further inquiries can be directed to the corresponding author.

## Author Contributions

CW and SZ conceived of the presented idea. ZD, LS, WS, and CW analyzed and wrote the manuscript. SZ contributed to critical revision of the manuscript. All authors discussed the results and contributed to the final manuscript.

## Conflict of Interest

The authors declare that the research was conducted in the absence of any commercial or financial relationships that could be construed as a potential conflict of interest.

## Publisher's Note

All claims expressed in this article are solely those of the authors and do not necessarily represent those of their affiliated organizations, or those of the publisher, the editors and the reviewers. Any product that may be evaluated in this article, or claim that may be made by its manufacturer, is not guaranteed or endorsed by the publisher.
